# Role of Telokin in Regulating Murine Gastric Fundus Smooth Muscle Tension

**DOI:** 10.1371/journal.pone.0134876

**Published:** 2015-08-10

**Authors:** Changlong An, Bhupal P. Bhetwal, Kenton M. Sanders, Avril V. Somlyo, Brian A. Perrino

**Affiliations:** 1 Department of Physiology & Cell Biology, Center of Biomedical Research Excellence, University of Nevada School of Medicine, Reno, Nevada, United States of America; 2 Department of Molecular Physiology and Biological Physics, University of Virginia, P.O. Box 800736, Charlottesville, Virginia, United States of America; Cinvestav-IPN, MEXICO

## Abstract

Telokin phosphorylation by cyclic GMP-dependent protein kinase facilitates smooth muscle relaxation. In this study we examined the relaxation of gastric fundus smooth muscles from basal tone, or pre-contracted with KCl or carbachol (CCh), and the phosphorylation of telokin S13, myosin light chain (MLC) S19, MYPT1 T853, T696, and CPI-17 T38 in response to 8-Bromo-cGMP, the NO donor sodium nitroprusside (SNP), or nitrergic neurotransmission. We compared MLC phosphorylation and the contraction and relaxation responses of gastric fundus smooth muscles from telokin^-/-^ mice and their wild-type littermates to KCl or CCh, and 8-Bromo-cGMP, SNP, or nitrergic neurotransmission, respectively. We compared the relaxation responses and telokin phosphorylation of gastric fundus smooth muscles from wild-type mice and *W/W* ^*V*^ mice which lack ICC-IM, to 8-Bromo-cGMP, SNP, or nitrergic neurotransmission. We found that telokin S13 is basally phosphorylated and that 8-Bromo-cGMP and SNP increased basal telokin phosphorylation. In muscles pre-contracted with KCl or CCh, 8-Bromo-cGMP and SNP had no effect on CPI-17 or MYPT1 phosphorylation, but increased telokin phosphorylation and reduced MLC phosphorylation. In telokin^-/-^ gastric fundus smooth muscles, basal tone and constitutive MLC S19 phosphorylation were increased. Pre-contracted telokin^-/-^ gastric fundus smooth muscles have increased contractile responses to KCl, CCh, or cholinergic neurotransmission and reduced relaxation to 8-Bromo-cGMP, SNP, and nitrergic neurotransmission. However, basal telokin phosphorylation was not increased when muscles were stimulated with lower concentrations of SNP or when the muscles were stimulated by nitrergic neurotransmission. SNP, but not nitrergic neurotransmission, increased telokin Ser13 phosphorylation in both wild-type and *W/W* ^*V*^ gastric fundus smooth muscles. Our findings indicate that telokin may play a role in attenuating constitutive MLC phosphorylation and provide an additional mechanism to augment gastric fundus mechanical responses to inhibitory neurotransmission.

## Introduction

Smooth muscle contraction and relaxation involves the phosphorylation and dephosphoryl-ation of the 20kDa regulatory light chain of myosin (MLC) by myosin light chain kinase (MLCK) and myosin light chain phosphatase (MLCP), respectively. Contraction is initiated by an increase in cytosolic Ca^2+^ ([Ca^2+^]_i_) and activation of Ca^2+^/CaM-dependent MLCK that phosphorylates MLC at S19. MLCP-dependent dephosphorylation of S19 moderates contractile force and eventually causes relaxation when [Ca^2+^]_i_ is restored to resting levels. Thus, contractile force is a function of the activity ratio between MLCK and MLCP. It is now clear that MLCP is a key signal processor under complex regulation. Decreasing the activity of MLCP leads to a phenomenon known as ‘Ca^2+^ sensitization’ in which a given increase in [Ca^2+^]_i_ can yield a greater level of MLC phosphorylation and contractile force [1, 2]. Ca^2+^ sensitization occurs through regulatory proteins, such as CPI-17 and MYPT1, that when phosphorylated by protein kinase C (PKC) or Rho kinase (ROCK), inhibit MLCP [1, 3–7].

In contrast to Ca^2+^ sensitization, stimuli that increase cAMP or cGMP levels, including nitric oxide (NO), atrial natriuretic factors, β-adrenergic agonists, and vasoactive intestinal peptide (VIP), can reduce Ca^2+^-sensitization, increase MLC dephosphorylation, and reduce contractile force. This process has been termed Ca^2+^ desensitization [6, 8–10]. In gastric fundus smooth muscles, as in most gastrointestinal (GI) smooth muscles, NO is the primary inhibitory neurotransmitter responsible for the relaxation underlying the gastric accommodation reflex [11]. The intracellular signaling events initiated by NO to relax smooth muscles are well known. NO binding to and activation of soluble guanylyl cyclase (GC) results in an increase in the cytosolic second messenger cGMP with concomitant activation of cGMP-dependent protein kinase (PKG) [12]. In smooth muscle cells (SMC), PKG activation opens the large conductance calcium-activated potassium channel (BKCa), inducing hyperpolarization of the membrane potential and a reduction in the calcium influx through voltage-dependent calcium channels [12]. In addition, phosphorylation of the inositol 1,4,5-triphosphate (IP_3_) receptor-associated PKG substrate (IRAG) inhibits calcium release from the sarcoplasmic reticulum [13]. Together, these cGMP-mediated mechanisms reduce cytosolic calcium levels and induce relaxation. However, there is still uncertainty as to how NO relaxes GI smooth muscles. This uncertainty is based on the question of whether SMCs or interstitial cells of Cajal (ICC) are the primary targets of NO released from enteric neurons. This question arises from the findings that both SMC and ICC express GC and PKG, and that in many regions of the GI tract, including gastric fundus, the ICC appear to be immediately adjacent to inhibitory neurons [14–16].

Telokin appears to be a signature regulatory protein involved in the relaxation responses of GI smooth muscles to NO [17]. Telokin is a smooth muscle-specific, 17-kDa protein that is transcribed from the same gene (MYLK1) that encodes smooth muscle MLCK, and its amino acid sequence is identical to the non-catalytic C terminal domain of smooth muscle MLCK [18–20]. Telokin, also known as Kinase-Related Protein (KRP), is independently transcribed through a promoter located within intron 28 of the MYLK gene, and is expressed at very high levels in intestinal, bladder, uterine, and portal vein smooth muscles, where its concentration is equivalent to the 52μM myosin head concentration [21]. Telokin is expressed at much lower levels in arterial smooth muscle [18, 22, 23]. Genetic deletion of telokin causes Ca^2+^ sensitization of ileum smooth muscle contraction, characterized by a decrease in MLCP activity and attenuated cGMP-induced relaxation [23]. In the present study we compared MLC S19 phosphorylation and the contractile and relaxation responses of gastric fundus smooth muscles from telokin^-/-^ mice and their wild-type littermates to exogenous agonists and cholinergic or nitrergic neurotransmission. We examined telokin S13 phosphorylation and the relaxation responses of gastric fundus smooth muscles to exogenous agonists or nitrergic neurotransmission. Investigating whether accessibility of NO to smooth muscles determines whether telokin phosphorylation is increased, we compared the relaxation responses and telokin S13 phosphorylation of gastric fundus smooth muscles from wild-type mice and *W/W* ^*V*^ mice to exogenous agonists or nitrergic neurotransmission. GI smooth muscle tissues of *W/W* ^*V*^ mice lack intramuscular interstitial cells of Cajal (ICC-IM), which are necessary to transduce the signal from neurally released NO to smooth muscle cells [24]. The overall aim of this study is to determine the importance of telokin in regulating both cGMP-induced relaxation via nitrergic stimulation, and the Ca^2+^ sensitivity of tonic smooth muscles of the gastrointestinal tract. Our findings indicate that telokin plays a role in Ca^2+^ sensitivity by attenuating constitutive MLC phosphorylation and provides an additional mechanism to augment gastric fundus mechanical responses to inhibitory neurotransmission.

## Materials and Methods

### Ethical approval

This study was carried out in strict accordance with the recommendations in the Guide for the Care and Use of Laboratory Animals of the National Institutes of Health. The protocol was approved by the University of Nevada, Reno Institutional Animal Care and Use Committee (Permit Number: 00424). Male C57BL/6J mice and *W/W* ^*v*^ mice were purchased from Charles River Labs (Hollister, CA, USA). Telokin^-/-^ mice were generated and characterized as previously described [23]. Because telokin is only expressed in smooth muscles, the effect of telokin deletion is specific to smooth muscles [23]. Mice at 6 to 8 weeks of age were used for all experiments. Mice were housed in a pathogen-free barrier facility on a 12-h light/dark cycle with free access to water and food (Prolab 5P76 Isopro 3000; 5.4% fat by weight). Euthanasia by cervical dislocation was performed under isofluorane anesthesia, and all efforts were made to minimize suffering.

### Tissue Preparation

Stomachs were removed, pinned to a Sylgard-lined dish containing 4°C oxygenated (97%O_2_/3%CO_2_) Krebs solution, the gastric fundus was identified and acquired, and the mucosa and submucosa removed by sharp dissection [25].

### Mechanical responses

Contractile activity was measured in static myobaths, with each gastric fundus smooth muscle strip (~10mm x 10mm) attached to a Fort 10 isometric strain gauge (WPI, Sarasota, FL, USA) in parallel with the circular muscles [26]. Solutions were changed every 30 min. A resting force of 0.6g was initially applied for optimal length-tension force development and the muscles were equilibrated for 1 h in 37°C oxygenated Krebs to allow each muscle strip to establish its own level of resting tension. The mechanical responses to exogenous compounds were measured from muscles incubated in the continuous presence of 0.3μM tetrodotoxin. The mechanical responses to neurally released NO were measured from muscles incubated with 100μM L-Nω-nitro-L-arginine (LNNA) and 1μM MRS2500, or 1μM atropine, 1μM phentolamine, and 1μM propranolol, respectively, for 20 min prior to the delivery of square wave pulses of electrical field stimulation (EFS) of 0.3 msec duration, 5–20 Hz, 150V, 30 sec duration (supra-maximal voltage; Grass S48 stimulator). Contractile responses were recorded and analyzed using Acqknowledge 3.2.7 software (BIOPAC Systems, Santa Barbara, CA, USA). At the indicated times during contraction the myobath chamber was rapidly dropped down and the tissue immediately submerged into ice cold acetone/10mM dithiothreitol (DTT)/ 10% (w/v) trichloroacetic acid (TCA) for 2 min, snap-frozen in liquid N_2_, and stored at -80°C for subsequent western blot analysis [26, 27]. EFS, was kept on while dropping the bath down and submerging the tissue into the ice cold acetone/DTT/TCA.

### SDS-PAGE and western blotting

Muscles were thawed on ice for 5 min, followed by three 1 min washes in ice cold acetone/DTT, and a 2 min wash in ice cold lysis buffer (mM; 50 Tris HCl pH 8.0, 60 β-glycerophosphate, 100 NaF, 2 EGTA, 25 Na-pyrophosphate, 1 DTT, with 0.5% NP-40, 0.2% SDS, and protease inhibitor tablet (Roche, Indianapolis, IA, USA)) [26, 27]. Each tissue was homogenized in 0.20 mL lysis buffer using a Bullet Blender (0.01% anti-foam C, 1 stainless steel bead per tube, speed 6, 5 min). The homogenates were centrifuged at 3000 x g, 4°C, 10 min, and the supernatants stored at -80°C. The supernatants were analyzed by SDS-PAGE and western blotting with rabbit anti-MYPT1 (sc-25618), rabbit anti-MLC (sc-15370), mouse anti-CPI-17 (sc-48406), and telokin antibodies, and phosphorylation levels were determined by western blot analyses using rabbit anti-pT696-MYPT1 (sc-17556-R), rabbit anti-pT853-MYPT1 (sc-17432-R), rabbit anti-pT38-CPI-17 (Santa Cruz Biotechnologies, CA, USA), and pS13-telokin anti-bodies. The preparation and characterization of mouse monoclonal anti-telokin and anti-pS13-telokin antibodies are previously described [23]. MLC phosphorylation was analyzed by phos-tag SDS-PAGE using 50μM phos-tag reagent and 100μM MnCl_2_, and western blotting with anti-MLC antibodies [28]. Protein bands were detected using horseradish peroxidase-conjugated goat-anti rabbit IgG (AP307P) or goat anti mouse IgG (AP181P) secondary antibodies (EMD Millipore, Billerica, MA, USA) and Lumigen TMA-6 (Lumigen, Southfield, MI, USA), and visualized with a CCD camera-based detection system equipped with Visionworks software (Epi Chem II, UVP Laboratory Products, Upland, CA, USA). The tiff images were inverted, and adjusted to auto levels and resolution with Adobe Photoshop (CS2, V9.0.2, Adobe Systems, San Jose, CA, USA) for densitometry [26].

### Statistical analysis

Protein concentrations were determined with the Bradford assay. Equal protein amounts were loaded for separation by SDS-PAGE. The densitometry values (pixel intensity units) of the protein bands were obtained using VisionWorks software. The densitometry values of total MYPT1, pT696, pT853, CPI-17, and pT38 were obtained from the protein doublets. The densitometry values of pS13, pT696, pT853, pT38, and pS19 were divided by the telokin, MYPT1, CPI-17, and MLC densitometry values, respectively, obtained from the same sample to obtain the ratio of phosphorylated protein to total protein. The ratios from control, untreated muscles were normalized to 1. For phos-tag analyzed MLC, the densitometry value of the upper (phosphorylated) MLC band was divided by the sum of the upper and lower (non-phosphoryl-ated) MLC bands to obtain the pS19:MLC ratio. Each independent experiment was analyzed with two independent Western blots. The ratios were analyzed by non-parametric repeated tests of ANOVA, and multiple comparisons within each experiment were analyzed with the Bonferroni test for significance using Prism 3.02 software, and are expressed as the averages ± SD. P<0.05 is considered significant. Contractile responses were measured by comparing the integrals, which measures the area under the curve including the contribution from the basal tone in a continuous fashion (amplitude units x horizontal units), per cross-sectional area (cm2) of the smooth muscles (gram-sec). Extents of relaxation from the basal tone (in grams) was also measured from the tone immediately prior to initiation of EFS-evoked relaxation to the level of tone at 30 sec for relaxation in response to EFS, or was measured from the tone immediately prior to addition of SNP or 8Br-cGMP, to the value of the tone at 1 min for SNP or 5 min for 8Br-cGMP. The percent relaxation of pre-contracted muscle strips was calculated by dividing the value of the tone in grams measured at 30 sec for relaxation in response to EFS or the value of the tone at 1 min for SNP or 5 min for 8Br-cGMP by the value of the tone in grams in the presence of KCl or CCh immediately prior to initiation of EFS-evoked relaxation or addition of the 8Br-cGMP or SNP, and then multiplying by 100. The average contraction and relaxation amplitudes and SD were obtained from the recordings and calculated using Prism 3.02 software and this data was analyzed by ANOVA. Multiple comparisons within each experiment were analyzed with the Bonferroni test for significance using Prism 3.02 software, and are expressed as the averages ± SD. *P*<0.05 is considered significant.

### Drugs

1H-[1,2,4]Oxadiazolo[4,3-a]quinoxalin-1-one (ODQ), 8-Bromo-cyclic GMP (8Br-cGMP), atropine, propranolol, phentolamine, and tetrodotoxin were purchased from EMD Millipore, Billerica, MA; L-NNA were obtained from Sigma-Aldrich, St. Louis, MO; MRS2500 was obtained from Tocris Bioscience, Minneapolis, MN; phos-tag was purchased from Wako Chemicals, Richmond, VA; and sodium nitroprusside was obtained from Fisher Scientific, Pittsburgh, PA.

## Results

### Telokin phosphorylation in non-pre-contracted gastric fundus smooth muscles is increased by agents that elevate cGMP

**Fig 1:**
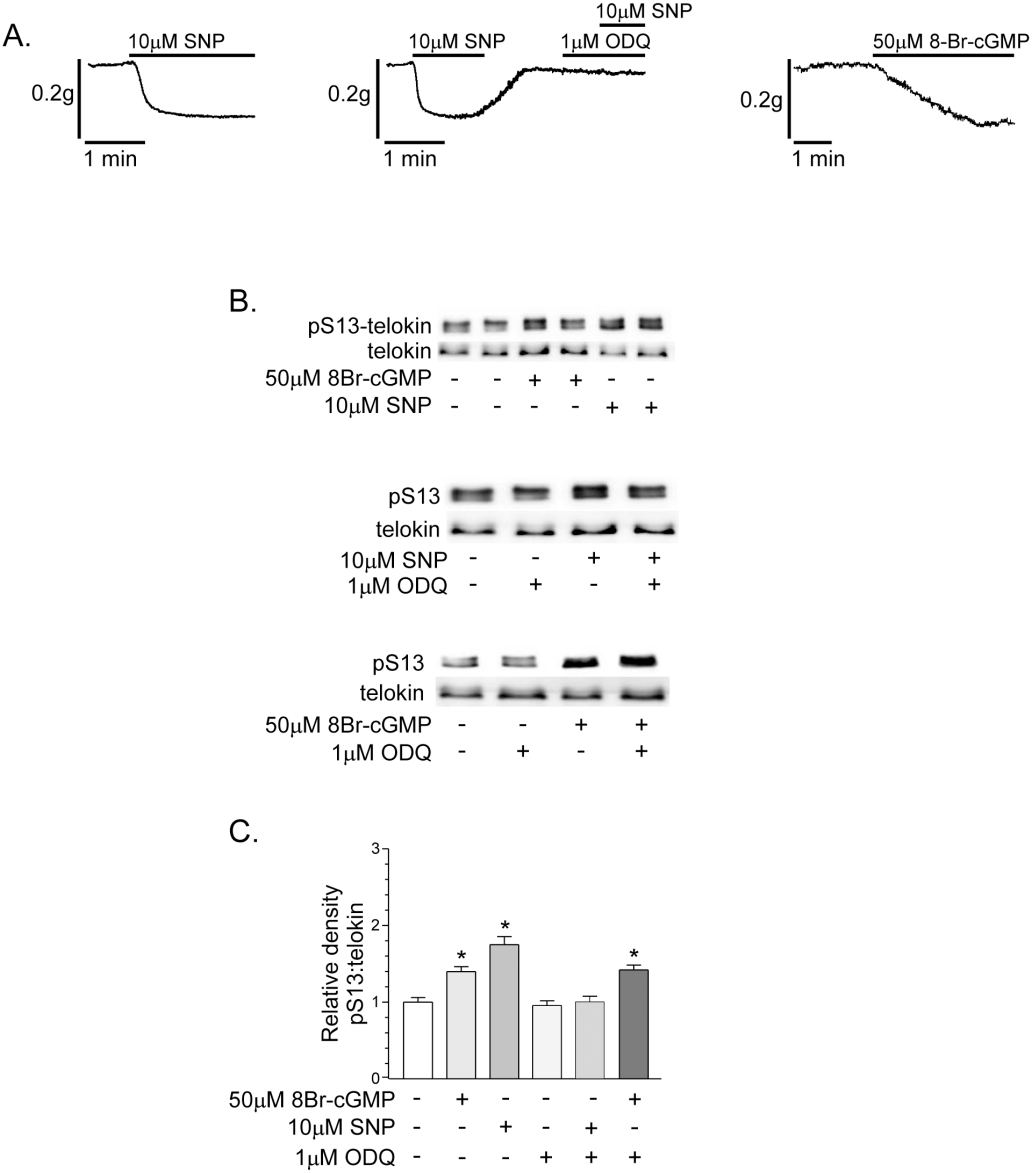
Telokin phosphorylation is increased by incubation of non-contracted gastric fundus smooth muscles with compounds that elevate cGMP. A. Representative recordings of the relaxation responses evoked by 10μM SNP in the absence or presence of 1μM ODQ (n = 6, each), or 50μM 8Br-cGMP (n = 6). B. Representative western blots of telokin S13 phosphorylation in untreated control muscles and muscles incubated with 50μM 8Br-cGMP for 5 min (n = 6) or 10μM SNP (n = 6) for 1 min. C. Average ratios ± SD of pS13:telokin in the absence or presence of 50μM 8Br-cGMP (n = 6) or 10μM SNP. *P<0.01 compared to untreated controls.

The basal tone of fundus muscle strips was reduced by 10μM SNP or 50μM 8Br-cGMP (Fig 1A). The maximum extents of relaxation to 10μM SNP and 50μM 8Br-cGMP were 0.16 ± 0.01g at 1 min, and 0.18 ± 0.007g at 5 min, respectively (n = 6, each). The GC inhibitor, ODQ (1μM), blocked the relaxation caused by SNP (Fig 1A). Telokin phosphorylations in fundus smooth muscles was measured before and after maximum levels of relaxation were reached, by SDS-PAGE and western blotting. At the levels of basal tone that develop in fundus muscles, telokin S13 phosphorylation was measured and normalized to 1 (i.e. 1.0 ± 0.17 intensity units; Fig 1B and 1C). Telokin S13 phosphorylation was increased 1.5 ± 0.18-fold and 1.75 ± 0.3-fold by 50μM 8Br-cGMP (n = 6) and 10μM SNP (n = 6), respectively (Fig 1B and 1C). The GC inhibitor, ODQ (1μM) blocked the increase in telokin S13 phosphorylation by SNP, but had no effect on the increase in telokin S13 phosphorylation evoked by 8Br-cGMP (Fig 1B and 1C, n = 6). In addition, ODQ alone had no effect on basal telokin S13 phosphorylation (Fig 1B and 1C, n = 6).

### Telokin phosphorylation in gastric fundus smooth muscles pre-contracted with 30mM KCl is increased by 8Br-cGMP or SNP

**Fig 2:**
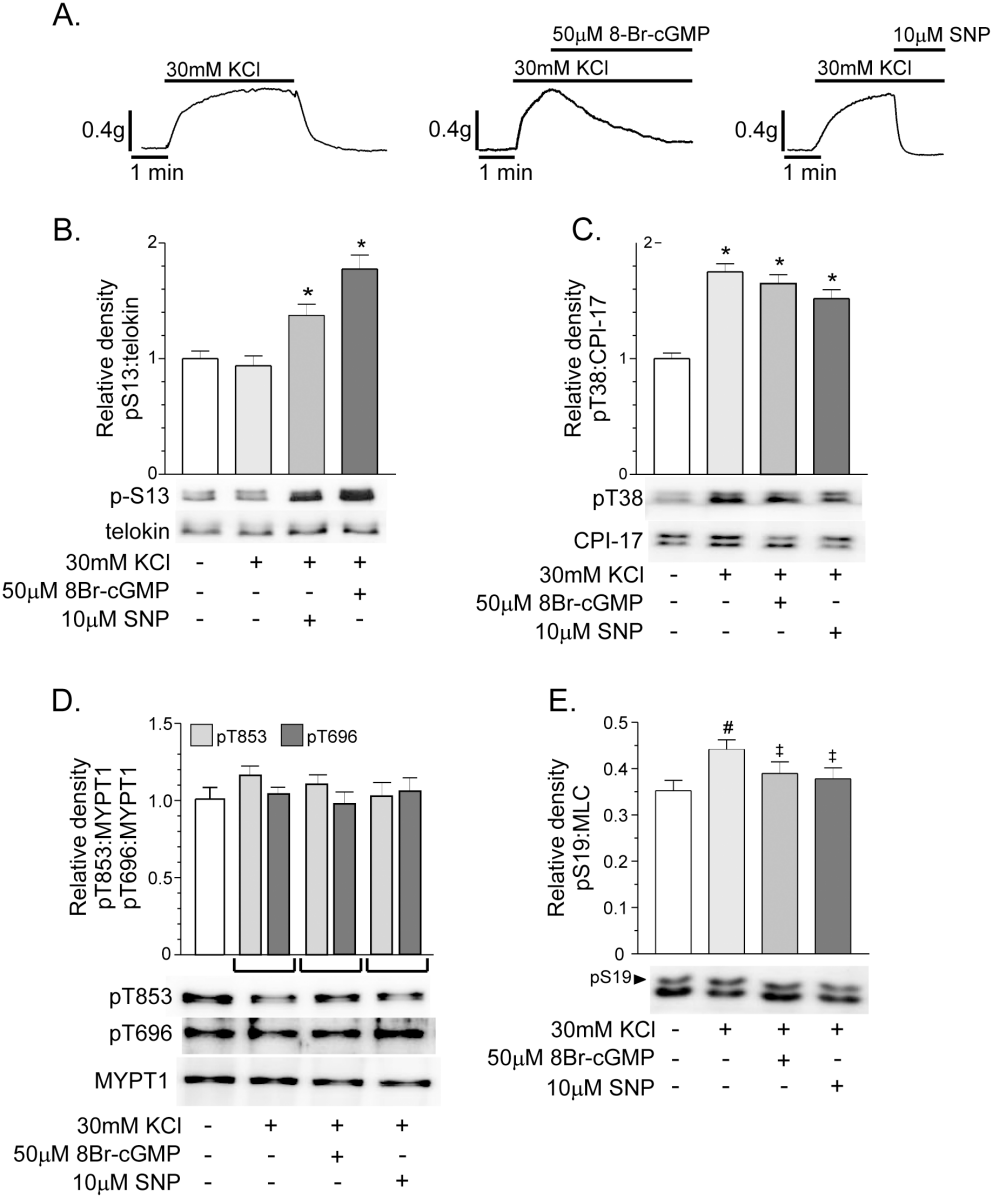
Telokin phosphorylation in gastric fundus smooth muscles pre-contracted with 30mM KCl is increased by compounds that elevate cGMP. A. Representative recordings of the contractile response to 30mM KCl, and the relaxation responses evoked by 50μM 8Br-cGMP (n = 6) or 10μM SNP (n = 6) in the presence of 30mM KCl. Average ratios ± SD of pS13:telokin, pT38:CPI-17, pT696:MYPT1, pT853:MYPT1, and pS19:MLC and representative western blots of the phosphorylation of (B) telokin S13, (C) CPI-17 T38, (D) MYPT1 T696 and T853, and (E) MLC S19 evoked by 50μM 8Br-cGMP (n = 6) or 10μM SNP (n = 6) in the presence of 30mM KCl. *P<0.01, ^#^ *P*<0.05, compared to untreated controls. ^‡^ *P*< 0.05 compared to 30mM KCl treated muscles.

We next examined the effects of cGMP elevating agents on fundus tone and telokin S13 phosphorylation using fundus smooth muscles pre-contracted with high K^+^. 8Br-cGMP (50μM) or SNP (10μM) increased telokin S13 phosphorylation and relaxed fundus smooth muscles pre-contracted with 30mM KCl (Fig 2). The percent relaxation evoked by 8Br-cGMP and SNP was 83 ± 3.3% and 100 ± 4.5%, respectively (n = 6). Telokin phosphorylation was increased 1.4 ± 0.2-fold and 1.8 ± 0.25-fold by SNP (n = 6) and 8Br-cGMP (n = 6), respectively (Fig 2B). High K^+^ alone had no effect on telokin S13 phosphorylation (Fig 2B) (n = 6), however CPI-17 T38 phosphorylation was increased 1.75 ± 0.14-fold by 30mM KCl (Fig 2C) (n = 6). CPI-17 phosphorylation remained elevated during the incubation of the muscle strips with 50μM 8Br-cGMP (1.65 ± 0.15) or 10μM SNP (1.52 ± 0.15) (Fig 2C). MYPT1 T696 and T853 phosphorylation was unaffected by 30mM K^+^ or by 8Br-cGMP and SNP (Fig 2D). At the peak of the high K^+^ contraction (2 min), MLC S19 phosphorylation was modestly, but significantly, increased (36 ± 3% vs. 44 ± 5% for control and 30mM KCl, respectively, n = 6) (Fig 2E). In the presence of 30mM KCl, 50μM 8Br-cGMP or 10μM SNP reduced MLC S19 phosphorylation back to the unstimulated levels (44 ± 5% vs. 39 ± 5%, and 37 ± 3% for 30mM KCl, 30mM KCl + 50μM 8Br-cGMP, and 30mM KCl + 10μM SNP, respectively) (Fig 2E). Thus, activation of telokin by 8Br-cGMP or SNP does not lead to a decrease in inhibitory phosphorylation of MYPT1 to account for the fall in KCl-induced force and MLC S19 phosphorylation.

### Telokin phosphorylation in gastric fundus smooth muscles pre-contracted with 1μM CCh is increased by 8Br-cGMP or SNP

**Fig 3:**
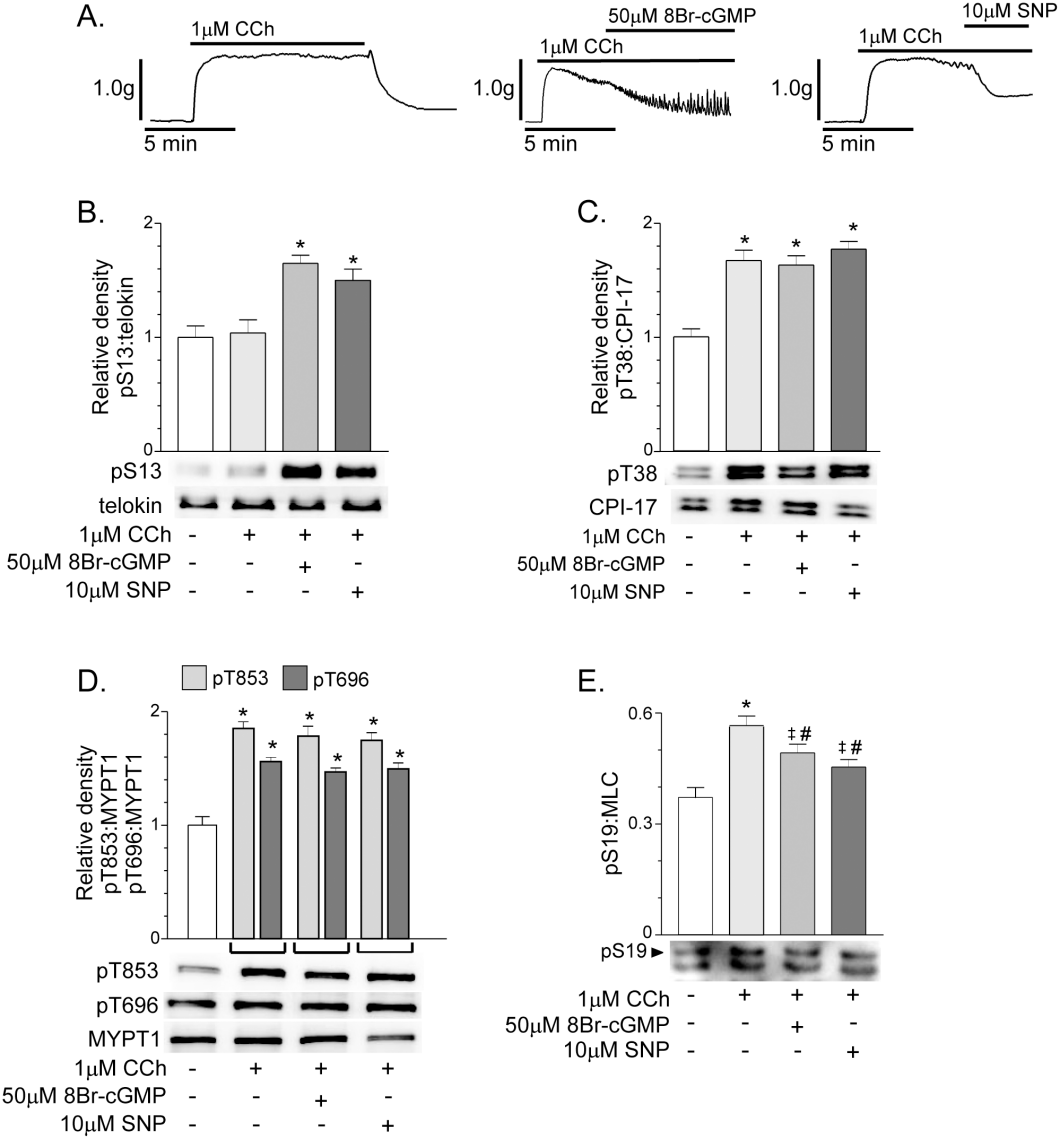
Telokin phosphorylation in gastric fundus smooth muscles pre-contracted with 1μM CCh is increased by compounds that elevate cGMP. A. Representative recordings of the contractile response to 1μM CCh, and the relaxation responses evoked by 50μM 8Br-cGMP (n = 6) or 10μM SNP (n = 6) in the presence of 1μM CCh. Average ratios ± SD of pS13:telokin, pT38:CPI-17, pT696: MYPT1, pT853:MYPT1, and pS19:MLC and representative western blots of the phosphorylation of (B) telokin S13, (C) CPI-17 T38, (D) MYPT1 T696 and T853, and (E) MLC S19 evoked by 50μM 8Br-cGMP (n = 6) or 10μM SNP (n = 6) in the presence of 30mM KCl. *P<0.01, ^#^ *P*<0.05, compared to untreated controls. ^‡^ *P*<0.05 compared to 1μM CCh treated muscles.

8Br-cGMP (50μM) or SNP (10μM) relaxed fundus smooth muscles pre-contracted with 1μM CCh and increased telokin S13 phosphorylation (Fig 3). The percent relaxation evoked by 8Br-cGMP and SNP was 77 ± 5.4% and 49 ± 3.6%, respectively (n = 6). Telokin phosphorylation was increased 1.65 ± 0.2-fold and 1.55 ± 0.14-fold by 8Br-cGMP and SNP, respectively (Fig 3B) (n = 6, each). CCh alone had no effect on telokin S13 phosphorylation (Fig 3B). CPI-17 T38 phosphorylation T38 (Fig 3C) and MYPT1 T696 and T853 phosphorylation (Fig 3D) were increased by 1μM CCh, as we previously reported [29]. The CCh-induced increases in T696, T853, and T38 phosphorylation were unaffected by 8Br-cGMP or SNP (Fig 3C and 3D). CCh (1μM/1min) significantly increased MLC S19 phosphorylation from 37 ± 5% to 56 ± 6% (Fig 3E). In the presence of 1μM CCh, 50μM 8Br-cGMP or 10μM SNP modestly, but significantly reduced MLC phosphorylation (56 ± 6% vs. 49±5%, and 46 ± 6% for 1μM CCh, 1μM CCh + 50μM 8Br-cGMP, and 1μM CCh + 10μM SNP, respectively), but these values were still higher than the control level (Fig 3E), possibly due to differences in the mechanisms underlying the force generated by high K^+^-induced depolarization and receptor-mediated contraction evoked by CCh.

### Telokin^-/-^ gastric fundus smooth muscles have increased contractile responses to exogenous agonists and to EFS-evoked cholinergic neurotransmission

**Fig 4:**
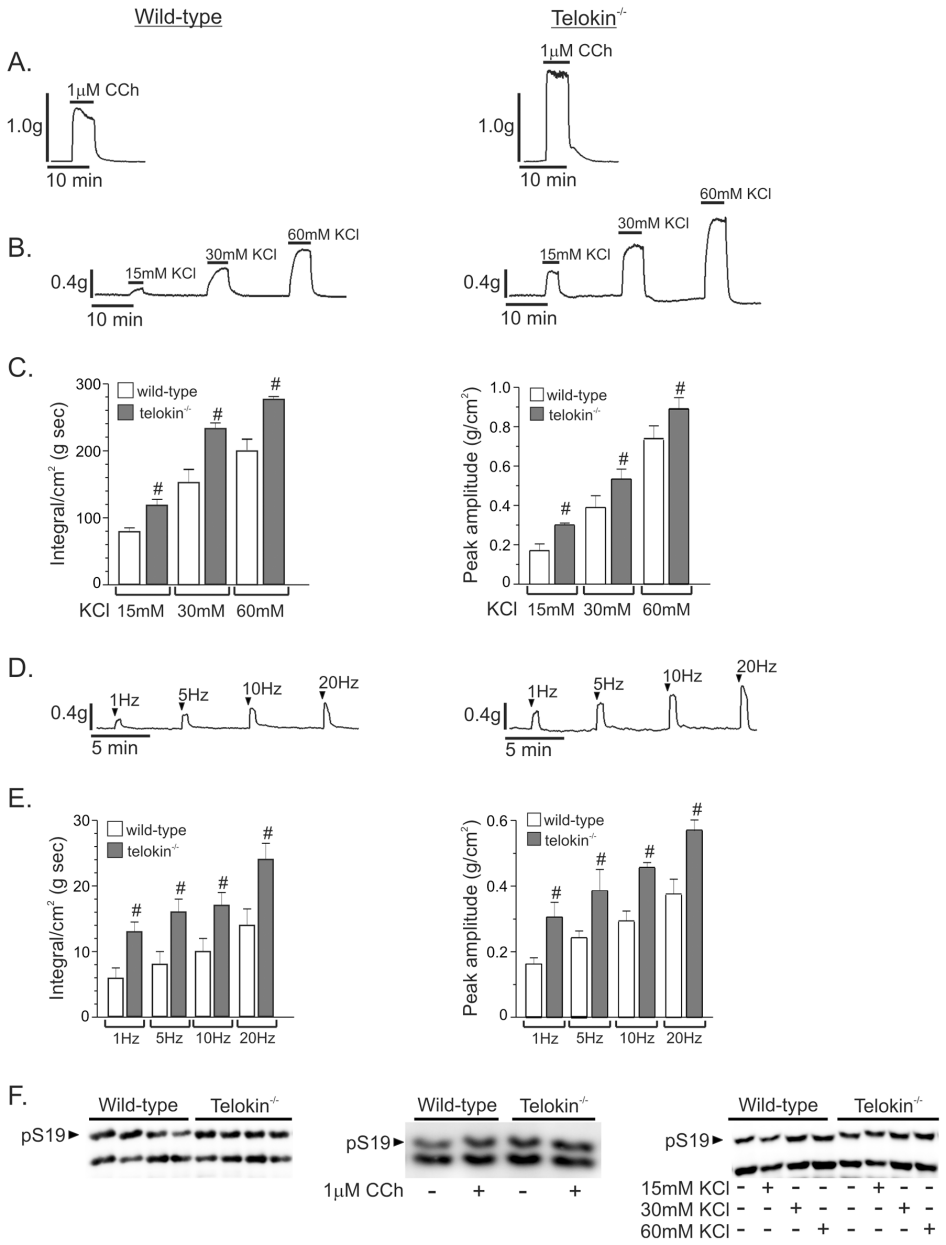
The contractile responses of telokin^-/-^ gastric fundus smooth muscles are increased compared to wild-type controls. A. Representative recordings of the contractile responses of wild-type and telokin^-/-^ gastric fundus smooth muscles to 1μM CCh (n = 4). B. Representative recordings of the contractile responses of wild-type and telokin^-/-^ gastric fundus smooth muscles to 15mM, 30mM, and 60mM KCl. C. Average integrals and peak amplitudes of contraction ± SD of wild-type and telokin^-/-^ gastric fundus smooth muscles to 15mM, 30mM, and 60mM KCl (^#^ *P*<0.05 compared to wild-type, n = 8). D. Representative recordings of the contractile responses of wild-type and telokin^-/-^ gastric fundus smooth muscles incubated with 100μM LNNA and 1μM MRS2500 to 30 sec of 1Hz, 5Hz, 10Hz, or 20Hz EFS. E. Average integrals and peak amplitudes of contraction ± SD of wild-type and telokin^-/-^ gastric fundus smooth muscles incubated with 100μM LNNA and 1μM MRS2500 to 30 sec of 1Hz, 5Hz, 10Hz, or 20Hz EFS KCl (^#^ *P*<0.05 compared to wild-type, n = 4). F. Representative western blots of phosphorylated and non-phosphorylated MLC in wild-type and telokin^-/-^ gastric fundus smooth muscles.

Since there was clear evidence of telokin phosphorylation in response to SNP and 8Br-cGMP, we tested the effects of loss of telokin on fundus contractile and relaxation responses by using telokin^-/-^ mice. Following the initial application of 0.6g of tension to muscle strips and equilibration at 37°C in oxygenated Krebs for 30-45min, telokin^-/-^ fundus muscle strips developed a higher level of resting tone than wild-type muscles (0.34 ± 0.06 g/cm^2^ vs. 0.18 ± 0.04 g/cm^2^, respectively. n = 4, *P*<0.01). In addition, the constitutive MLC S19 phosphorylation levels in telokin^-/-^ fundus smooth muscles were higher than the levels in wild-type muscles (49 ± 3% vs. 37 ± 2%, respectively, *P*<0.05, n = 4) (Fig 4F).

Contractile responses of telokin^-/-^ fundus muscles to 1μM CCh were significantly greater than the responses of wild-type muscles (Fig 4A) (n = 4). The mean integrals/cm^2^ of the contractile responses of wild-type and telokin^-/-^ fundus muscles to 1μM CCh were 215 ± 14 g sec and 422 ± 27 g sec, respectively (*P*<0.05, n = 4). The reduction in peak tone that typically occurred during sustained contraction to CCh was not observed with telokin^-/-^ fundus muscles (Fig 4A). As measured from the level of basal tone to the peak of the contractile response, the amplitudes of the contractile responses of telokin^-/-^ muscle strips to 1μM CCh were also larger than the responses of wild-type muscles (1.27 ± 0.2g vs. 0.89 ± 0.25g n = 4, *P*<0.05). The CCh-evoked increase in MLC S19 phosphorylation in telokin^-/-^ fundus smooth muscles was higher than the levels in wild-type muscles (62 ± 3% vs. 56 ± 6%, respectively, *P*<0.05, n = 4) (Fig 4F).

Contractile responses of telokin^-/-^ fundus muscles to 15mM, 30mM, and 60mM KCl were also greater than the responses of wild-type muscles (Fig 4B). The mean integrals of the responses of telokin^-/-^ fundus muscles to 15mM, 30mM, and 60mM KCl were each significantly higher than the mean integral values of wild-type muscles (Fig 4C). The mean amplitudes of the contractile responses from the basal tone level of telokin^-/-^ gastric fundus smooth muscle strips to 15mM, 30mM, and 60mM KCl were each also larger than the corresponding responses of wild-type muscles (Fig 4B and 4C). The increases in MLC S19 phosphorylation evoked by 30mM and 60mM KCl in telokin^-/-^ fundus smooth muscles were higher than the levels in wild-type muscles (52 ± 3% vs. 44 ± 5% for 30mM KCl, and 54 ± 4% vs. 47 ± 5% for 60mM KCl, Fig 4F, *P*<0.05, n = 4). MLC S19 phosphorylation was not significantly increased by 15mM KCl (Fig 4F, n = 4). Contractile responses of telokin^-/-^ fundus muscles evoked by 1Hz, 5Hz, 10Hz, and 20Hz (i.e. due to cholinergic neurotransmission) were also greater than the responses of wild-type muscles (Fig 4D). The mean integrals of the responses of telokin^-/-^ fundus muscles to EFS-evoked cholinergic neurotransmission were each about 2-fold higher than the mean integral values of wild-type muscles (Fig 4E). Finally, the amplitudes of the contractile responses of telokin^-/-^ fundus muscle strips to EFS were also each significantly larger than the corresponding responses of wild-type muscles (Fig 4D and 4E). As we previously reported, increased MLC phosphorylation in response to EFS was not detected (data not shown). Overall, these findings suggest that telokin-/- gastric fundus smooth muscles are more sensitive to excitatory contractile stimuli.

### Pre-contracted telokin^-/-^ gastric fundus smooth muscles have reduced relaxation responses to exogenous cGMP-elevating agents and nitrergic neurotransmission

**Fig 5:**
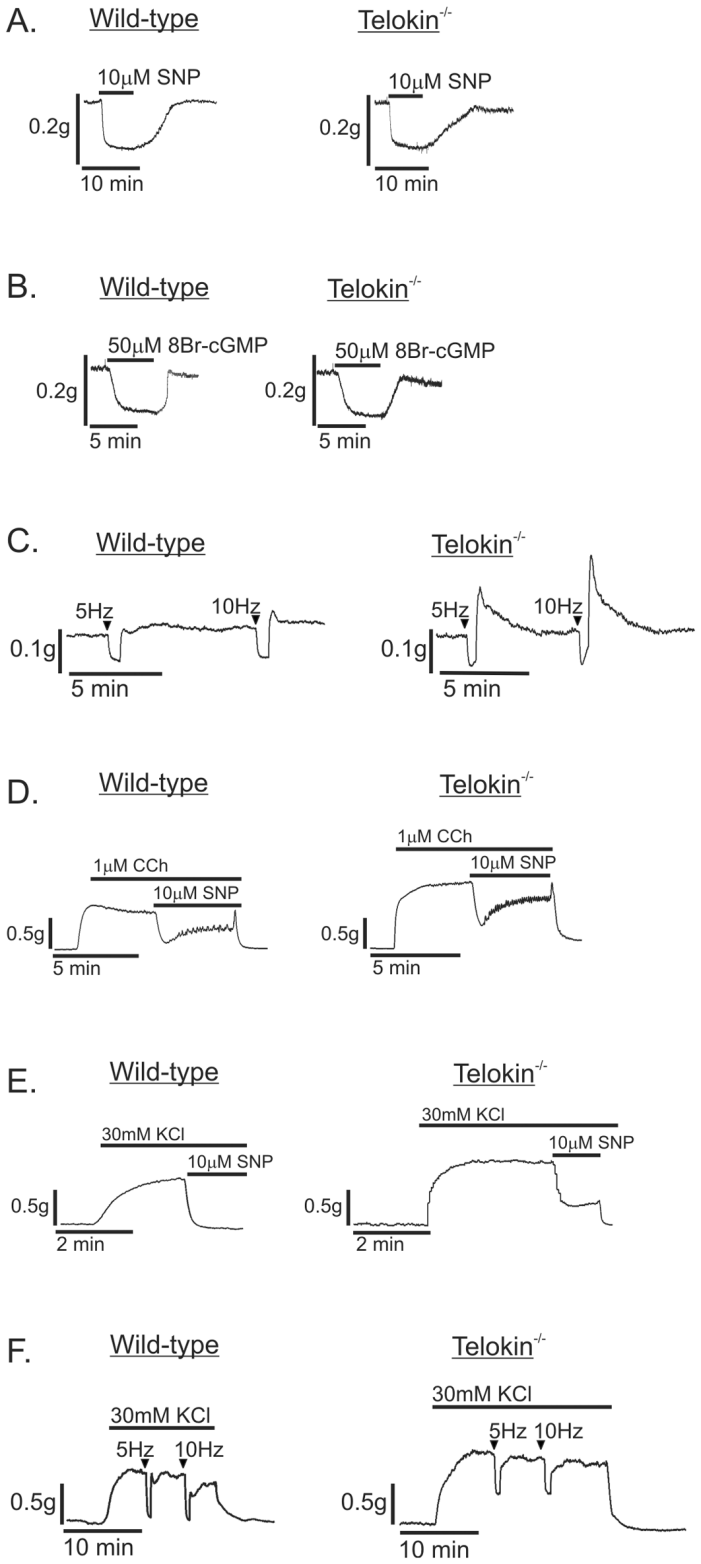
The relaxation responses of pre-contracted telokin^-/-^ gastric fundus smooth muscles are reduced compared to wild-type controls. Representative recordings of the relaxation responses of wild-type and telokin^-/-^ gastric fundus smooth muscles to (A) 10μM SNP, (B) 50μM 8Br-cGMP, and (C) 30 sec of 5Hz or 10Hz EFS. Representative recordings of the relaxation responses of wild-type and telokin^-/-^ gastric fundus smooth muscles evoked by 10μM SNP in the presence of (D) 1μM CCh or (E) 30mM KCl. F. Representative recording of the relaxation responses evoked by 30 sec of 5Hz or 10Hz EFS in wild-type and telokin^-/-^ gastric fundus smooth muscles incubated with 30mM KCl and 1μM atropine, 1μM phentolamine, and 1μM propranolol.

Similar extents of relaxation of non-pre-contracted wild-type and telokin^-/-^ muscles were observed in response to 10μM SNP (Fig 5A), 50μM 8Br-cGMP (Fig 5B), or 5Hz and 10Hz EFS-evoked nitrergic neurotransmission (Fig 5C) (n = 4, each). A larger post-stimulus contractile response (i.e. rebound contraction) was observed in telokin^-/-^ muscles (Fig 5C). The average amplitudes of relaxation of wild-type and telokin^-/-^ gastric fundus smooth muscles to 10μM SNP were 0.17 ± 0.01g and 0.15 ± 0.03g at 1 min, respectively (n = 4, *P*>0.05). Similarly, the average amplitudes of relaxation of wild-type and telokin^-/-^ fundus muscles to 50μM 8Br-cGMP were 0.18 ± 0.01g and 0.19 ± 0.01g at 5 min, respectively (n = 4, *P*>0.05). As shown in Fig 4, the amplitudes of contraction in response to CCh, high K^+^, or EFS were greater in telokin^-/-^ muscles vs. wild-type muscles. However, in these muscles pre-contracted by CCh (Fig 5D) or high K^+^ (Fig 5E), the amplitudes of relaxation evoked by SNP were reduced in telokin^-/-^ muscles vs. wild-type muscles. The amplitudes of relaxation were 86 ± 4.5% and 76 ± 2.5% of the peak amplitudes of contraction induced by 1μM CCh from wild-type and telokin^-/-^ gastric fundus smooth muscles, respectively (n = 4, *P*<0.05). The amplitudes of relaxation were 100 ± 7.3% and 72 ± 4.9% of the peak amplitudes of contraction induced by 30mM KCl from wild-type and telokin^-/-^ gastric fundus smooth muscles, respectively (n = 4, *P*<0.01). With muscles pre-contracted by high K^+^, the amplitudes of relaxation evoked by 5Hz and 10Hz EFS-evoked nitrergic neurotransmission (Fig 5F) were reduced in telokin^-/-^ muscles vs. wild-type muscles. The amplitudes of relaxation from 5Hz EFS were 83 ± 2.1% and 34 ± 3.2%, and from 10Hz EFS were 83 ± 2.7% and 29 ± 2.2% of the peak amplitudes of contraction induced by 30mM KCl from wild-type and telokin^-/-^ gastric fundus smooth muscles, respectively (n = 4, *P*<0.05).

### Telokin S13 phosphorylation is not increased by nitrergic neurotransmission

**Fig 6:**
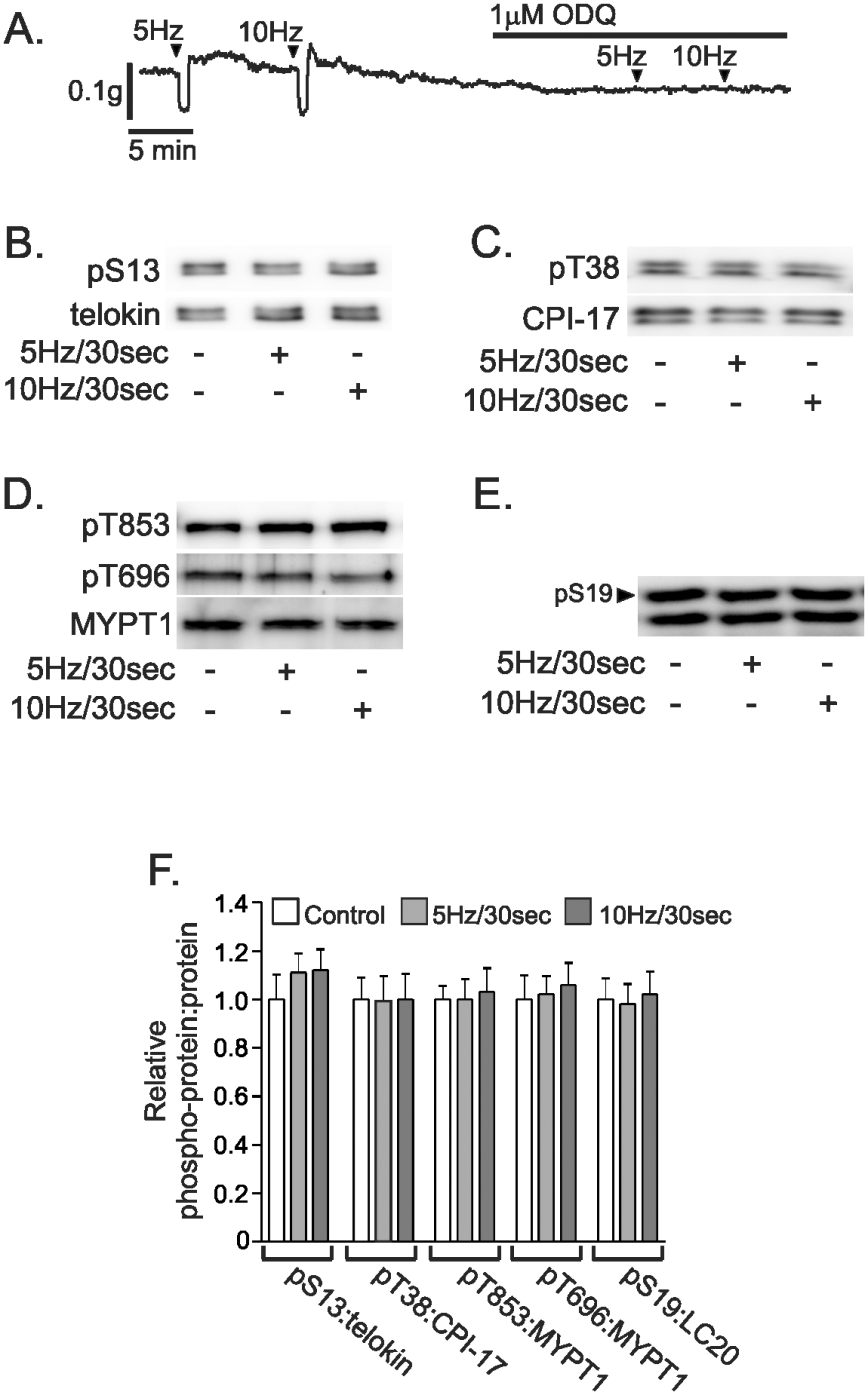
Telokin phosphorylation in non-contracted gastric fundus smooth muscles is not increased by nitrergic neurotransmission. A. Representative recording of the relaxation responses evoked by 30 sec of 5Hz or 10Hz EFS of muscles incubated in 1μM atropine, 1μM phentolamine, and 1μM propranolol. Representative western blots of the phosphorylation of telokin S13 (B), CPI-17 T38 (C), MYPT1 T853 and T696 (D), and MLC S19 (E) evoked by 30 sec of 5Hz or 10Hz EFS of muscles incubated in 1μM atropine, 1μM phentolamine, and 1μM propranolol. F. Average ratios ± SD of pS13:telokin, pT38:CPI-17, pT696:MYPT1, pT853:MYPT1, and pS19:MLC evoked by 30 sec of 5Hz or 10Hz EFS of muscles incubated in 1μM atropine, 1μM phentolamine, and 1μM propranolol (*P*>0.05, n = 6).

The effects of nitrergic neurotransmission on telokin phosphorylation were studied by EFS of wild type gastric fundus smooth muscle strips incubated with atropine, propranolol, and phentolamine to block excitatory motor inputs. Decreases in resting tone were measured in response to EFS (30 sec at 5Hz and 10Hz; Fig 6A). Responses to EFS were blocked by 0.3μM tetrodotoxin at the parameters of electrical stimulation used (data not shown). Maximum relaxation amplitudes of 0.07 ± 0.007g/cm^2^ and 0.08 ± 0.008g/cm^2^ were measured in response to 5Hz and 10Hz, respectively (*P*>0.05, n = 6). ODQ (1μM) blocked the relaxation responses, indicating that the responses were elicited by NO via synthesis of cGMP (Fig 6A). It should also be noted that treatment with ODQ did not unmask an excitatory component that enhanced contraction, suggesting that atropine effectively blocked excitatory neural inputs at these frequencies of stimulation. The muscle strips were processed for SDS-PAGE and western blotting after 30 sec of EFS. Constitutive telokin S13 phosphorylation did not increase in response to 5Hz and 10Hz EFS (Fig 6B). The phosphorylation of CPI-17 T38, MYPT1 T696, T853, and MLC S19 also were unchanged by stimulation under these circumstances (Fig 6C, 6D and 6E). Fig 6F shows the summarized data for the phosphorylation of telokin S13, CPI-17 T38, MYPT1 T696, T853, and MLC S19 in response to 5Hz and 10Hz EFS-evoked nitrergic neurotransmission.

### Telokin S13 phosphorylation is dose-dependently increased by SNP

**Fig 7:**
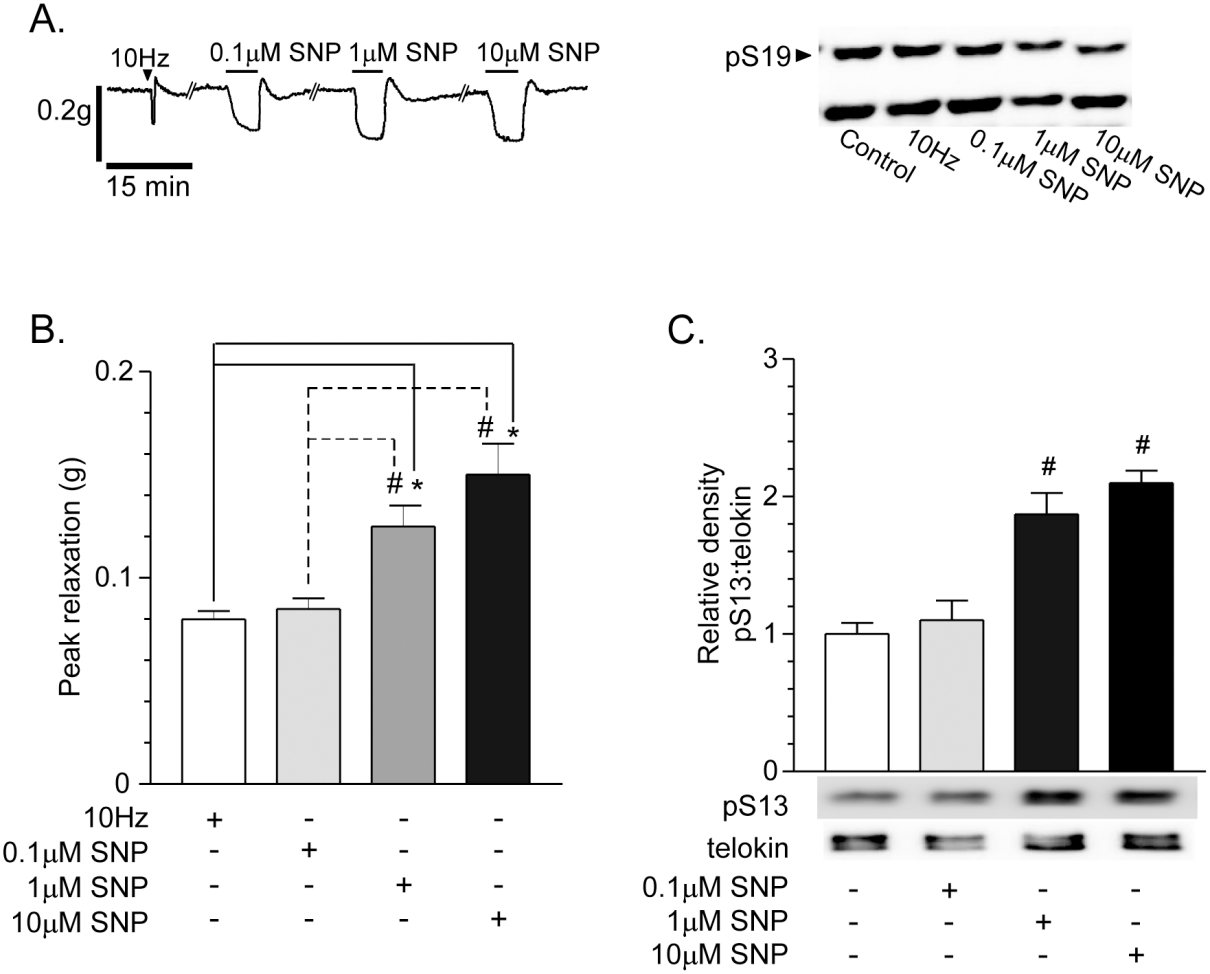
SNP dose-dependently increases telokin S13 phosphorylation in non-pre-contracted gastric fundus smooth muscles. A. Representative recording of the relaxation responses and representative western blot of phosphorylated and non-phosphorylated MLC evoked by 10Hz EFS, 0.1μM, 1μM, or 10μM SNP (n = 4). B. Average ratios ± SD of peak relaxation amplitudes evoked by 10Hz EFS, 0.1μM, 1μM, or 10μM SNP (*P<0.01, ^#^ *P*<0.05, n = 4). C. Average ratios ± SD and representative western blots of pS13:telokin evoked by 0.1μM, 1μM, or 10μM SNP (^#^ *P*<0.05 compared to 0.1μM SNP or 1μM SNP, n = 4).

Because we did not detect increases in telokin S13 phosphorylation in response to nitrergic neurotrans-mission, we determined whether telokin phosphorylation is coupled to the strength of the NO stimulus. In a dose-response study comparing the amplitudes of relaxation evoked by 10Hz/30sec nitrergic neurotransmission with SNP, we found that 1μM and 10μM SNP evoked significantly greater amplitudes of relaxation than 10Hz/30sec nitrergic neurotransmission or 0.1μM SNP (Fig 7A and 7B) (n = 4, each). Fig 7C shows that only 1μM and 10μM SNP in-creased telokin S13 phosphorylation. In addition, only 1μM and 10μM SNP decreased MLC S19 phosphorylation (Fig 7A, n = 4, Control, 37 ± 5%; 1μM SNP, 28 ± 5%; 10μM SNP, 28 ± 3%).

### Telokin S13 phosphorylation is not increased by nitrergic neurotransmission in *W/W* ^*V*^ fundus muscles

**Fig 8:**
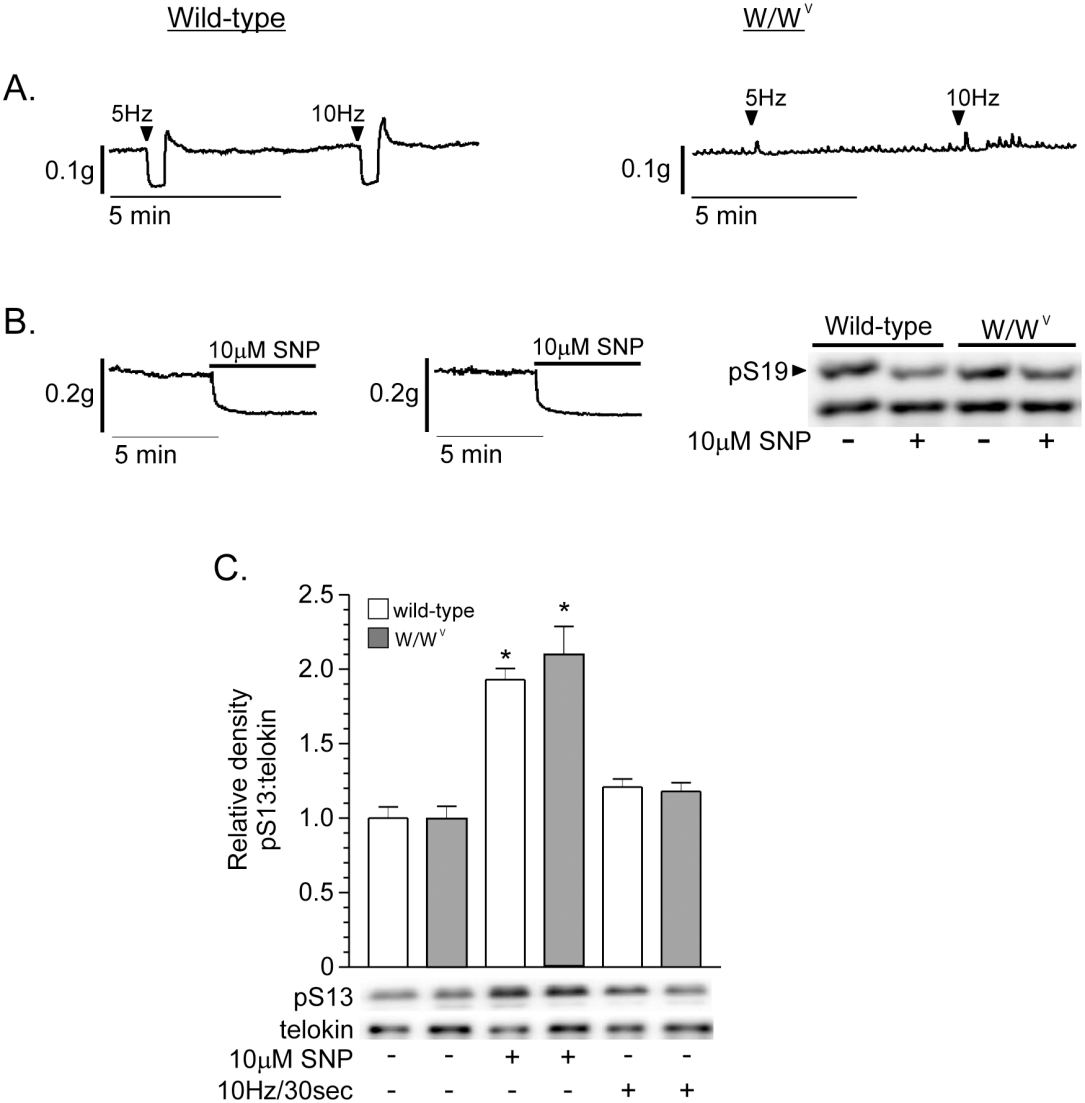
Telokin S13 phosphorylation is not increased by nitrergic neurotransmission in *W/W* ^*V*^ gastric fundus smooth muscles. Representative recordings of the relaxation responses evoked by 30 sec of 5Hz EFS and 10Hz EFS (A), or 10μM SNP (B) from wild-type and *W/W* ^*V*^ fundus muscles. B. Representative western blot of phosphorylated and non-phosphorylated MLC from wild-type and *W/W* ^*V*^ fundus muscles treated with 10μM SNP. C. Average ratios ± SD of pS13:telokin and representative western blots of phosphorylated telokin S13 and telokin from wild-type and *W/W* ^*V*^ muscles stimulated by 10Hz/30 sec EFS, or incubated with 10μM SNP. *P<0.01 compared to untreated wild-type or *W/W* ^*V*^ fundus muscle controls, n = 4.

The finding that telokin S13 phosphorylation was not increased by nitrergic neurotransmission, and was dose-dependently increased by SNP suggest that telokin phosphorylation is dependent upon access of NO to SMCs, and prompted us to examine telokin S13 phosphorylation in response to nitrergic neurotransmission in *W/W* ^*V*^ fundus muscle strips, which lack most ICC-IM [14]. Fig 8A shows that nitrergic neurotransmission relaxed wild-type fundus muscles, but relaxation was greatly decreased in *W/W* ^*V*^ fundus muscles, while Fig 8B shows that 10μM SNP relaxed both wild-type and *W/W* ^*V*^ fundus muscles (n = 6), as previously reported [14]. Fig 8C shows that telokin S13 phosphorylation in both wild-type and *W/W* ^*V*^ fundus muscles was not increased by nitrergic neurotransmission. However, SNP increased telokin S13 phosphorylation to similar extents in wild-type and *W/W* ^*V*^ fundus muscles. Telokin S13 phosphorylation was increased 1.85 ± 0.19-fold and 2.03 ± 0.3-fold by 10μM SNP in wild-type and *W/W* ^*V*^ fundus muscles, respectively (Fig 8C). 10μM SNP decreased MLC S19 phosphorylation to similar extents in wild-type and *W/W* ^*V*^ fundus muscles (Fig 8B, n = 6, wild-type, 37 ± 5%; *W/W* ^*V*^, 38 ± 6%; 10μM SNP, wild-type, 27 ± 5%; *W/W* ^*V*^, 10μM SNP, 28 ± 4%)

## Discussion

In this study we provide evidence that telokin provides a secondary mechanism for cyclic nucleotides to reduce Ca^2+^ sensitization and moderate gastric fundus smooth muscle mechanical responses to excitatory cholinergic stimulation. We found that telokin S13 is basally phosphoryl-ated and that the exogenous cyclic GMP-elevating agents 8-Bromo-cGMP and SNP increased the basal telokin phosphorylation. In muscles pre-contracted with KCl or CCh, 8-Bromo-cGMP and SNP had no effect on CPI-17 or MYPT1 phosphorylation, but increased telokin phosphorylation and reduced MLC phosphorylation. In telokin^-/-^ gastric fundus smooth muscles, basal tone and constitutive MLC S19 phosphorylation were increased. Pre-contracted telokin^-/-^ gastric fundus smooth muscles have increased contractile responses to KCl, CCh, or cholinergic neurotransmission and reduced relaxation to 8-Bromo-cGMP, SNP, and nitrergic neurotransmission. However, we could not resolve an increase in basal telokin phosphorylation when muscles were stimulated with lower concentrations of SNP or when the muscles were stimulated by activation of nitrergic motor neurons even though both forms of stimulation induced relaxation responses that reached 60% of the relaxation achieved by 10μM SNP. SNP, but not nitrergic neurotransmission, increased telokin Ser13 phosphorylation in both wild-type and *W/W* ^*V*^ gastric fundus smooth muscles. Thus, additional mechanisms likely contribute to nitrergic relaxation responses before recruitment of telokin to inhibit Ca^2+^ sensitization.

Khromov et al. reported that phosphorylated telokin associates with phosphorylated MYPT1, increasing its affinity for phosphorylated MLC resulting in a fall in MLC phosphorylation and force with no detectable change in MYPT1 phosphorylation at T696 and T853 [30]. We previously found high constitutive phosphorylation levels of MYPT1 at T696 and T853 in murine gastric fundus smooth muscles [26]. In the present study we showed there is constitutive phosphorylation of telokin at S13. In vitro studies show that MLCP is still partially active when MYPT1 is phosphorylated at T696 and T853 [31, 32]. In addition, we found that the MLCK inhibitor wortmannin reduces constitutive MLC phosphorylation [26]. Together with these previous findings, the present study shows that telokin is also constitutively phosphorylated suggesting a mechanism to set a particular level of MLCP activity in unstimulated gastric fundus smooth muscles and sustain the dynamic equilibrium between the MLCP and MLCK activities maintaining constitutive MLC phosphorylation. In addition, these findings raise the question as to the mechanism maintaining constitutive telokin phosphorylation in gastric fundus smooth muscles. Elevated basal cGMP levels that buffer excitatory pathways as reported in SMCs in cerebral blood vessels may also occur in fundus SMCs to account for the measured basal phosphorylated telokin [33].

We found that exogenous application of the cGMP-elevating compounds SNP and 8Br-cGMP to the myobath solution relaxed non-pre-contracted gastric fundus smooth muscles and increased telokin S13 phosphorylation. The extents of relaxation and the levels of increased telokin S13 phosphorylation induced by SNP or 8Br-cGMP were similar, although the kinetics of relaxation were different, presumably reflecting different rates of crossing cell membranes and mechanisms of elevating intracellular cGMP levels. SNP induced maximum extents of relaxation of non-precontracted gastric fundus smooth muscles within 1 min of exposure, while the maximum extent of relaxation by 50μM 8Br-cGMP was attained after 5 min. Both 8Br-cGMP and SNP increased telokin S13 phosphorylation between 1.5- and 2-fold above constitutive levels. SNP and 8Br-cGMP also induced similar increases in telokin S13 phosphorylation of approximately 1.5-fold in gastric fundus smooth muscles pre-contracted by either 1μM CCh or 30mM KCl. CCh or high KCl alone did not increase telokin phosphorylation. As we previously reported, 1μM CCh increased MYPT1 and CPI-17 phosphorylation [29], and these increases were not affected during the relaxation and increase in telokin phosphorylation evoked by SNP or 8BrcGMP. In addition, 30mM KCl increased CPI-17, but not MYPT1 phosphorylation, and the increased CPI-17 phosphorylation was not affected during the relaxation and increase in telokin phosphorylation evoked by SNP or 8Br-cGMP. These findings are consistent with previous reports that telokin does not reverse the inhibitory phosphorylation of MYPT1 at T696 and T853 [30]. In addition, the present findings provide the first indication that the inhibitory phosphoryl-ation of CPI-17 at T38 is also unaffected by telokin phosphorylation.

Tone in the fundus is dependent upon Ca^2+^ entry through a voltage-dependent pathway, although the channels utilized may differ between species. In canine fundus, dihydropyridines had little effect on [Ca^2+^]_i_ in fundus muscles, but lemakalim (K_ATP_ agonist that induced hyperpolarization) and Ni^+^ decreased tone significantly [34]. In the murine fundus, nicardipine reduce the force of contractile responses to CCh by about 50% [29]. Previous studies have shown that NO donors hyperpolarize fundus muscles [14], and this could result in a decrease in [Ca^2+^]_i_ and tone. The conductance activated by NO that caused hyperpolarization appeared to reside in ICC, because hyperpolarization responses to NO donors was greatly reduced in *W/W* ^*V*^ muscles lacking ICC [14]. Nevertheless, relaxation responses to NO donors persisted in *W/W* ^*V*^ muscles suggesting that mechanisms in addition to hyperpolarization can mediate nitrergic relaxation. In the present study we confirmed that relaxation responses to NO donors persisted in *W/W* ^*V*^ muscles and telokin phosphorylation increased in these muscles. Nitrergic relaxation responses to EFS, however were greatly reduced in *W/W* ^*V*^ muscles, suggesting that changes in [Ca^2+^]_i_ may linked to ICC membrane potential responses in the mouse.

Similar to the findings from a previous study of ileum smooth muscle, we found that the contractile responses of telokin^-/-^ gastric fundus smooth muscles to CCh, high KCl, or neurally released ACh were greater than the contractile responses of wild-type muscles [23]. Much of the enhanced contractile response may be related to the increased basal tone in telokin^-/-^ muscles, as the increases in peak amplitudes were modest, although once elevated, the CCh-evoked sustained tone of telokin^-/-^ muscles remained elevated, in contrast to wild-type muscles. In addition, constitutive MLC S19 phosphorylation was elevated in telokin^-/-^ fundus muscles. These findings suggest that telokin phosphorylation may have a role in modulating the contractile responses of gastric fundus smooth muscles to excitatory stimuli by attenuating the level of constitutive MLC phosphorylation. It should be noted that in addition to increasing the activity of MLCP, *in vitro* studies suggest that telokin can inhibit MLC phosphorylation by MLCK and Ca^2+^-independent MLC kinases [35]. However, it is not clear that these *in vitro* studies recapitulate the intracellular environment in terms of the concentrations and organization of the contractile machinery. Furthermore, earlier studies demonstrated that telokin did not inhibit the rate of MLC phosphorylation by ATPγS nor did it slow the rate or change the amplitude of the Ca^2+^-induced contraction when MLCP activity was inhibited [17, 36]. However, we cannot rule out the possibility of some inhibitory role for telokin in the gastric fundus. Without telokin to enhance activity of MLCP or inhibit MLC kinases, constitutive MLCP activity may be lower and constitutive MLC kinase activity may be higher than normal in telokin^-/-^ muscles. These differences may shift the balance between kinase and phosphatase activities toward increased MLC phosphorylation, causing enhanced basal tone in telokin^-/-^ muscles. In addition, in muscles contracted by CCh, we noticed that typical decline in tone following the peak contraction was reduced in telokin^-/-^ muscles. In contractile responses involving G_q_-coupled receptors in GI smooth muscles, following the initial MLCK-dependent contraction the sustained contraction reaches a dynamic equilibrium dependent upon the balance between MLC kinase and MLCP activities [37]. The sustained MLC phosphorylation may be due to staurosporine-sensitive, Ca^2+^-independent kinases, including zipper-interacting protein (ZIP) kinase and integrin-linked kinase [38–40]. The pathways that lead to inhibition of MLCP by G_q11/12/13_-coupled receptors involve the sequential activation of G_q11/12/13_, RhoGEF, RhoA and ROCK-mediated MYPT1 phosphoryl-ation, and/or PKC-mediated CPI-17 phosphorylation [37]. Without telokin to activate MLCP and inhibit MLC kinases, MLCP activity is presumably decreased and MLC kinase activity is presumably increased during the sustained phase of contraction in telokin^-/-^ muscles, shifting the balance between kinase and phosphatase activities in favor of kinase activity and more sustained force during receptor-mediated contraction.

The amplitudes of relaxation of non-pre-contracted telokin^-/-^ gastric fundus smooth muscles in response to EFS-evoked nitrergic neurotransmission were similar to those of wild-type muscles, although the tension rebound was larger at the termination of the stimulus. These results are consistent with the findings that EFS-evoked nitrergic neurotransmission did not increase telokin S13 phosphorylation in non-pre-contracted wild-type fundus muscles. We expected the amplitudes of relaxation of telokin^-/-^ fundus muscles evoked by 8Br-cGMP and SNP to be less than those of wild-type muscles because we found that 8Br-cGMP or SNP increased telokin phosphorylation and relaxed non-pre-contracted wild-type muscles. However, we found that the relaxation responses of telokin^-/-^ fundus muscles to SNP or 8Br-cGMP were diminished compared to wild-type control muscles only when the muscles were pre-contracted with CCh or 30mM KCl. The reduced relaxation of pre-contracted telokin^-/-^ fundus muscles may be due to an additive effect due to the loss of MLCP activation by phosphorylated telokin, shifting the balance between kinase and phosphatase activities in favor of kinase activity during both the contraction and relaxation responses. We found that MLC S19 phosphorylation was, to some extent, negatively associated with telokin S13 phosphorylation. Although nitrergic neurotrans-mission relaxed non-pre-contracted fundus muscles, telokin S13 phosphorylation was not increased and MLC S19 phosphorylation was not decreased. However, the increased S19 phosphorylation induced by CCh or high K^+^ were partially reversed as 8Br-cGMP and SNP relaxed the muscles and telokin S13 phosphorylation increased. Also, constitutive S19 phos-phorylation was slightly, but significantly, higher in telokin^-/-^ fundus muscles than wild-type muscles. These findings suggest that modulation of MLCP activity by telokin phosphorylation contributes to gastric fundus relaxation by reducing MLC phosphorylation.

The amplitudes of relaxation of non-pre-contracted fundus muscles evoked by 1μM or 10μM SNP were greater than those evoked by ODQ-sensitive 10Hz EFS or 0.1μM SNP, and only 1μM and 10μM SNP increased telokin S13 phosphorylation. Because telokin expression is exclusive to SMCs [19], these findings led us to conclude that accessibility of NO to smooth muscle GC determines whether telokin phosphorylation is increased. To test this conclusion we examined the effects of NO on telokin phosphorylation and the relaxation responses of gastric fundus smooth muscles from wild-type and *W/W* ^*V*^ mice. The exogenous NO donor SNP evoked similar relaxation responses in wild-type and *W/W* ^*V*^ gastric fundus muscles. In contrast, as shown previously, *W/W* ^*V*^ fundus muscles were much less responsive to nitrergic neurotransmission evoked by 5Hz or 10Hz EFS. These findings indicate that, although NO from exogenous NO donors can relax gastric fundus smooth muscles by directly accessing SMCs, but ICC are necessary for SMC relaxation in response to NO released from motor neurons. These results provide additional evidence for dual or multiple pathways for nitrergic relaxation mediated by GC in ICC, and/or GC in SMC as reported in murine fundus lacking GC β1 subunit in ICC, SMC or both [41]. These findings also provide an explanation for our findings showing that nitrergic neurotransmission, 0.1μM, 1μM, and 10μM SNP relaxed gastric fundus muscles, but only 1μM and 10μM SNP resulted in increased telokin phosphorylation (Fig 7).

In conclusion we examined an exogenous NO donor, exogenous cyclic nucleotide elevating agonists, and nitrergic neurotransmission on telokin S13 phosphorylation and relaxation responses. Exogenous cyclic GMP-elevating agents, but not nitrergic neurotransmission, increased telokin S13 phosphorylation above the significant basal levels, reduced MLC phos-phorylation and decreased tone in gastric fundus smooth muscles. Nitrergic neurotransmission inhibited tone but telokin phosphorylation was not affected. Investigating whether accessibility of NO to smooth muscle GC determines whether telokin phosphorylation is increased; we found that the exogenous NO donor SNP, but not nitrergic neurotransmission, increased telokin phos-phorylation and relaxed *W/W* ^*V*^ gastric fundus muscles. We compared the contractile and relax-ation responses and MLC S19 phosphorylation of fundus smooth muscles from telokin^-/-^ and wild-type mice to exogenous agonists and endogenous cholinergic or nitrergic neurotrans-mission. We found that telokin is constitutively phosphorylated and that its phosphorylation is increased in resting or pre-contracted gastric fundus smooth muscles by an exogenous NO donor or by cyclic nucleotide elevating agents. We suggest that the basal level of phosphorylated telokin also contributes to recovery of tone following nitrergic-induced relaxation. We found that telokin^-/-^ fundus muscles have elevated basal tone and MLC S19 phosphorylation, and increased contractile responses to exogenous agonists and to ACh released from neurons. We also found that relaxation to an NO donor and neurally released NO is reduced in pre-contracted telokin^-/-^ fundus muscles. The findings from this study suggest that telokin is not a required factor for nitrergic relaxation in the fundus, but it might be recruited to permit maximal contraction in the presence of high concentrations of NO, or may play a role in attenuating constitutive MLC phosphorylation by regulating the constitutive activity of MLCP and contributing to rapid recovery from nitrergic stimulation.
